# *APOE4* genotype exacerbates the depression-like behavior of mice during aging through ATP decline

**DOI:** 10.1038/s41398-021-01631-0

**Published:** 2021-10-05

**Authors:** Wenting Fang, Naian Xiao, Guirong Zeng, Daode Bi, Xiaoman Dai, Xue Mi, Qinyong Ye, Xiaochun Chen, Jing Zhang

**Affiliations:** 1 Department of Neurology and Geriatrics, Fujian Institute of Geriatrics, Fujian Medical University Union Hospital, 29 Xinquan Road, Fuzhou Fujian, 350001 China; 2 Fujian Key Laboratory of Molecular Neurology, Institute of Neuroscience, Fujian Medical University, 88 Jiaotong Road, Fuzhou Fujian, 350005 China; 3 Department of Neurology, The First Affiliated Hospital of Xiamen University, 55 Zhenhai Road, Xiamen Fujian, 361003 China

**Keywords:** Depression, Neuroscience

## Abstract

Population-based studies reveal that apolipoprotein E (*APOE*) **ε**4 gene allele is closely associated with late-life depression (LLD). However, its exact role and underlying mechanism remain obscure. The current study found that aged apoE4-targeted replacement (TR) mice displayed obvious depression-like behavior when compared with age-matched apoE3-TR mice. Furthermore, apoE4 increased stress-induced depression-like behaviors, accompanied by declines in the hippocampal 5-HT (1A) radioligand [18F] MPPF uptake evidenced by positron emission tomography (PET). In [18F]-fluorodeoxyglucose PET ([18F]-FDG PET) analyses, the FDG uptake in the prefrontal cortex, temporal cortex and hippocampus of apoE4-TR mice significantly declined when compared with that of apoE3-TR mice after acute stress. Further biochemical analysis revealed that ATP levels in the prefrontal cortex of apoE4-TR mice decreased during aging or stress process and ATP supplementation effectively rescued the depression-like behaviors of elderly apoE4-TR mice. In primary cultured astrocytes from the cortex of apoE-TR mice, apoE4, when compared with apoE3, obviously decreased the mitochondrial membrane potential, mitochondrial respiration, and glycolysis in a culture time-dependent manner. Our findings highlight that apoE4 is a potential risk factor of depression in elderly population by impairing the glucose metabolism, reducing ATP level, and damaging mitochondrial functions in astrocytes, which indicates that in clinical settings ATP supplementation may be effective for elderly depression patients with apoE4 carrier.

## Introduction

It has been a winding road to identify genomic loci potentially associated with depression by genome-wide association studies (GWAS) and candidate loci studies. In humans, three major apolipoprotein E isoforms (*APOE2*, *APOE3*, and *APOE4*) have been documented and extensive epidemiological and genetic screening has revealed a possible association between apolipoprotein E (*APOE*) polymorphism and depression. Notably, from the age perspective, *APOE* **ε**4 allele can potentially identify people at high risk for clinically significant depression [1–5], forwarding the onset age of late-life depression (LLD) [6], and is associated with severer depressive symptoms [7]. Moreover, in stressful circumstances, *APOE* **ε**4 allele carriers are at an increased risk of mental illness [8, 9]. On the contrary, some studies suggest that there is no association between *APOE* **ε**4 allele and depression [10]. Therefore, further studies are needed to confirm the relationship between the APOE ε4 allele and depression. In the central nervous system (CNS), apoE is mainly secreted by astrocytes, with different isoforms playing distinct roles in lipid metabolism and nervous system homeostasis [11, 12]. Studies have shown that *APOE4* increases the sensitivity of cells to oxidative stress and that apoE4 (1-272) fragment appears to interact directly with mitochondria, resulting in mitochondrial dysfunction and neurotoxicity [13]. However, it remains obscure how *APOE4* increases the risk of LLD.

One essential pathophysiological feature of depression is energy metabolism disorders [14–16]. PET examination has reported decreased blood flow and impaired glucose metabolism in the caudate nucleus, anterior cingulate cortex, and prefrontal cortex of patients with depression [14, 17]. PET studies have also revealed that human carriers of the *APOE* **ε**4 allele experience reduced brain glucose metabolism at a young age (20–39 years old) [18]. In the depression mice model, features of abnormal energy metabolism have also been documented, including abnormal glucose metabolism, elevated lactate levels, mitochondrial dysfunction, and abnormal ATP levels [19–22]. Under hypoxia, cerebrovascular accidents, highly-activated neurons, and other stress conditions, astrocytes release ATP, which provides energy for neuron activity [23]. Studies have shown that insufficient ATP release in astrocytes leads to depression-like behaviors, while stimulation of ATP release in astrocytes produces an antidepressant effect [24]. Given that astrocytic atrophy and decreased expression of the astrocyte biomarker GFAP have been documented in both animal depression models and autopsy specimens of depressed patients [25], we propose that the impaired *APOE4* genotype-dependent glucose metabolism may induce depression susceptibility during stress or aging.

In this study, we investigated the depression-like behaviors in apoE4-TR mice at varied ages, examined the 5-HT and glucose metabolism by PET-CT imaging, and studied mitochondrial respiration and glycolysis in primary cultured astrocytes with a Seahorse XF96 extracellular flux analyzer. We found that the *APOE4* genotype significantly promoted the occurrence of depression-like behaviors during aging. Further detection of ATP concentration showed that ATP levels in the prefrontal cortex of apoE4-TR mice decreased during aging or stress processes. Of note, the administration of ATP effectively ameliorated the depression-like behaviors of elderly apoE4-TR mice. In vitro experiments evidenced that the *APOE4* genotype and extended culture time damaged the mitochondrial membrane potential, mitochondrial respiration, and glycolysis of primary astrocytes. Our results demonstrate that *APOE4* is a risk factor for depression during aging. Impaired glucose metabolism and ATP decline of apoE4-TR mice contribute to increased depression-like behaviors.

## Materials and methods

### Animals

The human apoE-TR homozygous male mice with the C57BL/6J background were purchased from Taconic (www.taconic.com), in which the expression of human *APOE3* or *APOE4* is controlled by the mouse *APOE* promoter, replacing the endogenous mouse *APOE* [26]. All animals were maintained on a 12-h light/dark cycle in standard plastic cages (6 mice/cage) with food and water available *ad libitum*. All experiments were approved by the Medical Animal Care and Use Committee of Fujian Medical University and observed *The Guide for the Care and Use of Laboratory Animals* by the U.S. National Institutes of Health (NIH Publications No. 80-23, revised in 1996). Genomic DNA was isolated by treating mousetail with proteinase K. A 227 bp sequence of the apoE gene was amplified by PCR using primers. Genotyping was performed after digestion with the restriction enzyme *Hha*1 and separation of fragments on a 12% polyacrylamide gel [27].

### Forced swim test (FST)

**Fig. 1:**
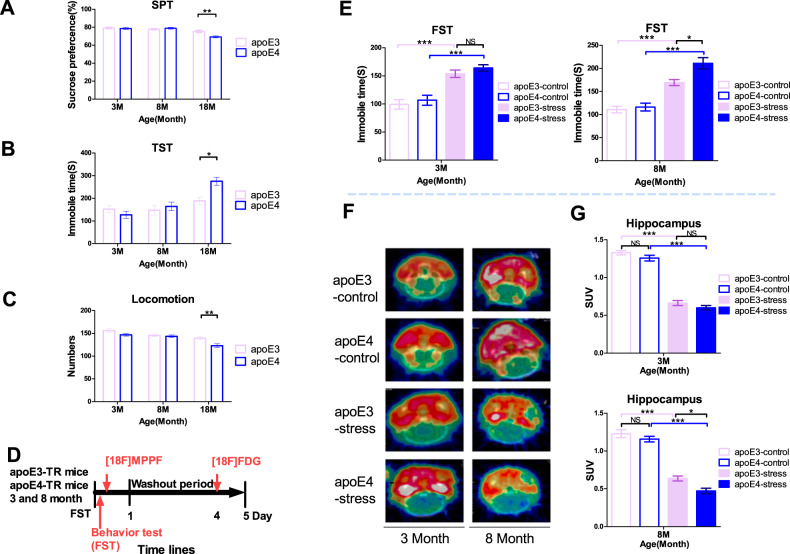
The *APOE* ε4 allele-induced increase in depression-like behaviors in an age-dependent manner. Depression-like behavior measurements of apoE3-TR and apoE4-TR mice at 3, 8, and 18 months of age, including sucrose consumption percentage in SPT (**A**), immobility time in the TST (**B**), locomotion in the open field (**C**). *p < 0.05, **p < 0.01, by two-way ANOVA with Tukey’s Multiple Comparisons Test (*n* = 13–16). **D** The time line of FST and behavioral test of apoE3-TR and apoE4-TR mice at 3, 8 months of age. **E** The immobility time in the forced swim test (FST). *p < 0.05, **p < 0.01, ***p < 0.001, by two-way ANOVA with Tukey’s Multiple Comparisons Test (*n* = 8–13). **F** The reconstruction of PET-CT images demonstrated a remarkable MPPF uptake in the hippocampus. **G** Hippocampus standard intake value (SUV) chart, *p < 0.05, **p < 0.01, ***p < 0.001, by two-way ANOVA with Tukey’s Multiple Comparisons Test (*n* = 7–8). NS not significant (*p* > 0.05). Data are presented as the mean ± S.E.M.

Based on reference [28], the mice received two consecutive swimming sessions in a glass cylinder (height: 35 cm; diameter: 20 cm) containing water (depth: 25 cm; temperature: 16 °C). On the first day, the mice underwent a 15-min forced swimming test (training session) in the cylinder; 24 h later, they received a 6-min forced swimming test (test session). The immobility time in the 6-min test was recorded manually. The time schedule was depicted in Fig. 1D.

### ATP supplementation experiment

ATP (Sigma, USA) was dissolved with saline (NS). The dose of ATP (125 mg/kg/day) was adjusted according to previous studies [24]. Briefly, ATP or saline was intraperitoneally injected once a day for 7 days. Animals were divided into saline treatment group and ATP supplement group according to the random number table.

### Behavioral tests

#### Sucrose preference

Before the sucrose preference experiment, the mice were trained to drink water and sucrose water freely in two bottles whose positions were switched every day and the daily sucrose water consumption was recorded for one week. The sucrose preference experiment commenced when a stable sucrose water consumption was evident. In brief, after a 24-h period of water fasting, the animals were allowed free access to two bottles containing water and 1% sucrose solution, respectively, for two hours. Sucrose preference was calculated as the ratio of sucrose water intake to the total volume of liquid intake. In the behavioral tests, we used double-blind tests.

#### Tail suspension test (TST)

Mice were suspended 28 ± 2 cm above the floor with adhesive tape placed ~1 cm from the tip of the tail. In the experiment (Fig. 1B, E), immobility time was manually counted. In the ATP supplement experiment, the immobility time in the 6-min test was recorded by Shanghai Xinsoft Behavioral Software (Shanghai Xinsoft, China). In the behavioral tests of two batches of animals, we used double-blind tests.

#### Open field test (OFT)

Each mouse was placed in the center of an open box (50 × 50 × 50 cm in size), and the area was divided into nine squares of 16.67 cm × 16.67 cm. In the experiment (Fig. 1C), vertical movement (rearing and exploratory behavior) for 10 min was manually counted. In the ATP supplement experiment, vertical movement was recorded by Shanghai Xinsoft Behavioral Software (Shanghai Xinsoft, China). In the behavioral tests of two batches of animals, we used double-blind tests.

#### Radiosynthesis of [18F] MPPF and [18F] FDG

The synthesis of [18F] MPPF was performed according to the procedure reported previously [29]. In brief, no-carrier-added [18F] fluoride was produced by proton bombardment of enriched 18O-water via the 18O (p, n)18F reaction, and was then trapped in an SEP-PAK LIGHT QMA (Waters). The [18F] fluoride was eluted from the cartridge into a reaction vessel with a K2.2.2./K2CO3 solution (2-mL 96/4 MeCN/water, 9.5-mg K2.2.2., 1.7-mg K2CO3). The elute was dried at 100 °C under a gentle stream of nitrogen gas. Then, 10 mg of benzamide, N-{2-[4-(2-methoxyphenyl)-1-piperazinyl] ethyl}-4-nitro-N-2-pyridinyl, in anhydrate DMSO (1 mL) was added, and the mixture was then heated at 140 °C for 20 min. The reaction was then quenched and purified by semipreparative high-performance liquid chromatography (HPLC), at a mobile phase [60% 50 mM NaOAc (pH = 5), 15% THF, 25% MeOH (v/v); flow rate: 5 mL/min]. The fraction corresponding to [18F] MPPF was collected into the round bottomed dilution flask and simultaneously diluted into sterile water. This solution was then passed through a Waters C18 Sep-Pak to trap the [18F] MPPF while the acetonitrile left over from the HPLC buffer was washed away. Following trapping, the cartridge was rinsed with sterile water (12 mL). [18F] MPPF was then eluted into the collection vial with ethanol (0.5 mL) and diluted with sterile saline (9.5 mL). The dose was then passed through a sterile filter into a sterile vial and used for QC testing. Typical yields of [18F] MPPF using this method were 13.2 ± 5.9% (*n* = 5).

[18F] FDG was manufactured in accordance with the standard method described by our laboratory using the coincidence [18F] FDG synthesis module (TracerLab FxFN, GE Healthcare). Quality control of the radiosynthesis was performed by HPLC with a radiochemical purity of 95%.

#### MicroPET/CT imaging

[18F]-MPPF and [18F]-FDG imaging was obtained by Siemense Inveon PET/CT. The mice were injected with about 24–26 MBq of [18F] MPPF via the tail vein. At 15 min p.i., the mice were anesthetized with isoflurane (5% for induction and 2% for maintenance in 100% O_2_) using a knockdown box. With the help of a laser beam attached to the scanner, the mice were placed in the prone position near the center of the FOV of the scanner. Static microPET images (5 min) were obtained after the whole-body CT scan (6 min). The images were reconstructed using ordered subset expectation maximization with three-dimensional resolution recovery (OSEM 3D) with CT-based attenuation correction and scatter correction. For data analysis, the region of interest (ROI) was manually drawn on the CT images, and the ROI was copied to the corresponding PET images. The mean standard uptake value (SUV mean) of the ROIs were recorded.

Two days after the [18F] MPPF acquisition, the microPET imaging acquisition of [18F] FDG was performed 40 min after the injection of about 18.5–29.6 MBq of [18F] FDG via the tail vein.

#### ATP content measurement

Before ATP evaluation, the tissue or cell was sonicated in a cold lysis buffer (containing 0.1 M PBS, 1 M Na3VO4, 2 mM NaF, 2.5 mM Na4P2O7, 1% Triton X-100 and 1% protease inhibitor cocktail). Then, supernatants were collected by centrifuging at 16,000 × *g* for 25 min at 4 °C. Protein concentration was determined with a BCA protein assay kit according to the manufacturer’s protocol (Thermo Scientific, USA). The ATP content was measured with a Microplate Reader and the ATP Bioluminescence Assay Kit HS II (Roche Molecular Biochemicals, Germany) according to the manufacturer’s instructions. ATP concentration was determined by FlexStation 3, a multifunctional marker made by Molecular Devices (MD) inc.

In brief, the ATP standard curve determination was performed as follows: the ATP standard solution was first diluted to the appropriate concentration gradient with ATP detection lysate. Then the ATP test working fluid was prepared by diluting an appropriate amount of ATP detection reagent at a ratio of 1:100. The ATP concentration was then determined: 50 μl of ATP detection working solution was added to the detection wells and 50 μl sample was added to 96-well black detection plates. An equal volume of 50 μl of luciferase reagent was added to each well. The standard ATP and sample were duplicated in 2 wells. A fluorescent microplate reader was immediately employed to determine the fluorescence intensity. The parameters were set as follows: well scan; pattern: cross; density: 3; spacing: 1:1.13 mm; total points: 5; luminescence integration: 25; top read; lm1 all; Automix: once; calibrate: on; PMT: auto; settle time: on; read/well: 1; RLU Min: 0; RLU Max: 20000. Finally, the concentration of ATP in the sample was calculated from the standard curve. The standard concentration (nmol/mg) was calculated on the basis of protein concentration.

#### Primary astrocyte culture

The newborn mice were sterilized with alcohol and separated to obtain brain tissue. The meninges and vascular membranes were then removed. The isolated brain tissue was transferred to a centrifuge tube containing a salt solution (Salt solution main component: NaCl 137 mM, KCl 5.3 mm, MgCl2 1 mM, Glucose 25 mM, HEPES 10 mM, CaCl2 3 mM) with 20 U/ml papain (Worthington, Lakewood, NJ), 0.5 mM EDTA, 0.2 mg/ mL L-cysteine and a small amount of DNA enzyme, and digested in a 37 °C water bath for 20 min. The digestion was then stopped and washed twice with an astrocyte culture medium (main component: F-12 medium) (HyClone, Utah, USA), 10% FBS (HyClone, Utah, USA), Penicillin-Streptomycin Solution (HyClone, Utah, USA). Then, the culture medium was refreshed. The tissue was dispersed with a pipette and then inoculated into a polylysine-coated flask.

#### Flow cytometry analysis of JC-1 staining

The astrocytes at the bottom of the flask were digested with trypsin, which was terminated in the F12 medium containing 10% FBS. A cell suspension (5 × 10^5^ cells/ml) was prepared by blowing through a straw. The mitochondrial membrane potential was measured using a Flow Cytometry Mitochondrial Membrane Potential Detection Kit (BD™ MitoScreen) according to the instruction manual. First, 1 ml of cell suspension was transferred into a sterile polystyrene centrifuge tube (15 ml). The cells were then centrifuged at 400 × *g* for 5 min at room temperature and the supernatant was carefully removed and discarded. Freshly prepared JC-1 working solution (0.5 ml) was added to each tube. Cells in the JC-1 working solution were gently resuspended and incubated at 37 °C in a CO_2_ incubator for 15 min. Afterwards, 1× assay buffer (1 ml) was administered to each tube. The cells were subsequently gently resuspended and centrifuged at 400 × *g* for 5 min. Finally, each cell pellet was gently resuspended in 0.5 ml of 1× assay buffer and analyzed by flow cytometry.

#### Measurement of mitochondrial respiration and glycolysis

Mitochondrial respiration and glycolysis were measured by oxygen consumption (OCR) and extracellular acid rate (ECAR) with a Seahorse XF96 extracellular flux analyzer (Seahorse Bioscience). Cortical astrocytes from apoE3-TR and apoE4-TR mice were cultured for both 1 and 2 months and subsequently digested with trypsin and re-inoculated into Seahorse XF96 plates at a density of 10,000 cells per well. OCR and ECAR tests were performed two days later.

In brief, on the day of the mitochondrial stress test, the astrocytes were washed three times with a medium (XF Base Medium, 10 mM glucose, 2 mM glutamine, 1 mM sodium pyruvate; pH adjusted to 7.4 with NaOH). Afterwards, the astrocytes were incubated at 37 °C without CO_2_ for one hour before determination. Changes of cellular respiration were evaluated over time after the injection of 1 μM oligomycin in port A, 0.5 μM FCCP in port B, and 0.5 μM rotenone/antimycin in port C. For mitochondrial measurements, the following parameters were determined: basal respiration (average OCR of three measurements preceding oligomycin injection) and maximum respiration (average OCR of four measurements between FCCP and before rotenone/antimycin A injection).

The glycolytic stress test was performed according to the Seahorse Bioscience specification. In brief, the cell plate was washed three times with the medium (XF Base Medium, 2 mM glutamine, and pH adjusted to 7.4 with NaOH) on the day of the test. Afterwards, the cells were incubated in a CO_2_ free incubator at 37 °C for 1 h. Continuous injection of glucose (10 mM, port A), oligomycin (1mu, port B), and 2-deoxyglucose (50 mM, port C) was employed in the glycolytic stress tests. For glycolysis, the following parameters were determined: glycolysis rate (average ECAR of four measurements between glucose and oligomycin injection) and glycolysis capacity (average ECAR of four measurements between oligomycin and 2-deoxyglucose injection).

### Statistical analysis

Data were analyzed with SPSS 22.0 statistical software and quantitative data were expressed as mean ± SEM. All the charts were produced with GraphPad Prism 5.03 software. Appropriate statistical tests were chosen depending on the experimental conditions. The details were specified in the legends. Statistical significance was set at *p* < 0.05.

## Results

### *APOE* ε4 allele mediates depression-like behaviors in mice in an age-dependent manner

ApoE-TR mice at different ages (3 months, 8 months, 18 months) underwent a series of depression-like behavior tests to investigate whether the *APOE* ε4 allele is a risk factor of depression. Between apoE3-TR mice and apoE4-TR mice at 3 months old and 8 months old, no significant difference was found in either the sucrose preference test (SPT), immobility time in tail suspension test (TST), or the total number of rearings in the open field test (OFT) (Fig. 1A–C). Of note, compared with age-matched apoE3-TR mice, the 18-month-old apoE4-TR mice showed a lower sucrose consumption, longer immobility time in TST, and fewer rearings (Fig. 1A–C).

As stress increases the susceptibility to depression, we further investigated whether the *APOE* ε4 allele may aggravate the susceptibility of mice to stress by subjecting apoE-TR mice to forced swimming test (an acute depression animal model). After FST stress, the immobility time of both 3-month-old and 8-month-old apoE3-TR and apoE4-TR mice increased (Fig. 1E). Given the same stress, the immobility time was significantly longer in the 8-month-old apoE4-TR mice than in age-matched apoE3-TR mice (Fig. 1E), but no similar effect was found between those 3-month-old apoE-TR mice. The results indicate that middle-aged apoE4-TR mice are more sensitive to stress-induced depression than age-matched apoE3-TR mice.

Abnormalities in neurotransmitters, especially in monoamine neurotransmitters, are the main pathophysiological features of depression and the main target for current antidepressant treatments. Among them, 5-HT tryptophan and its receptor are widely recognized and used as biochemical indicators of depression. In the 5-HT receptor family, 5-HT1A is the most important receptor associated with depression. Highly affinitive with the 5-HT1A receptor is [18F] MPPF radiolabel, first reported in 1997 [30]. As expected, FST treatment obviously decreased the [18F]-MPPF SUV mean values in the hippocampus of both apoE3-TR mice and apoE4-TR mice. Of note, in the FST treatment groups, the [18F]-MPPF SUV value of the 8-month-old apoE4-TR mice significantly declined when compared with that of age-matched apoE3-TR. However, no similar effect was observed in 3-month-old apoE-TR mice (Fig. 1G). The behavioral results were further confirmed by PET examination, which showed that the level of the neurotransmitter 5-HT, closely related to the occurrence of depression, significantly decreased in the brain of apoE4-TR mice when compared with that of apoE3-TR mice. Altogether, the results evidence that the *APOE* ε4 allele increases the sensitivity to depression-like behaviors under aging and stress.

### *APOE* ε4 allele induces glucose metabolism decline in the frontal cortex, temporal and hippocampus cortex of mice in an age-dependent manner

**Fig. 2:**
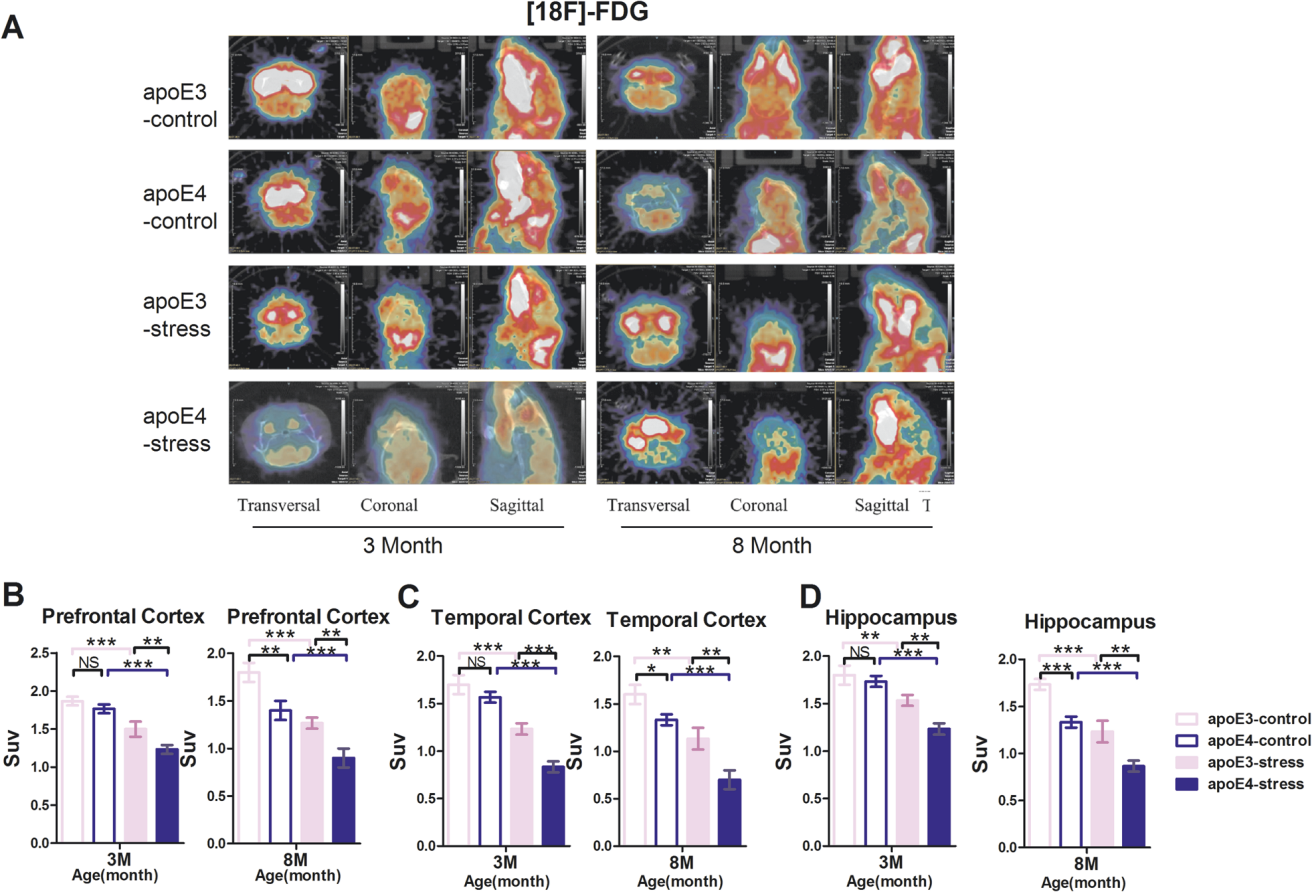
The *APOE* ε4 allele-induced glucose metabolism declines in the prefrontal cortex and the temporal cortex in an age-dependent manner. **A** The reconstruction of PET-CT images demonstrated a remarkable [18F] FDG uptake in the prefrontal cortex and temporal cortex. **B**–**D** Standard intake value (SUV) chart of the prefrontal cortex, temporal cortex and hippocampus, respectively. *p < 0.05, **p < 0.01, ***p < 0.001, by two-way ANOVA with Tukey’s Multiple Comparisons Test (*n* = 3). NS not significant (*p* > 0.05). Data are presented as the mean ± S.E.M.

The reconstruction of PET-CT images demonstrated a remarkable [18F]-FDG uptake. As shown in Fig. 2A, after FST, the [18F]-FDG uptake in the whole brain of mice was lower than that of age-matched control mice. As the differences of [18F]-FDG uptake in the certain brain region between FST and control mice cannot be visually detected and there are variations in the injected doses and animal weights, the standard uptake value (SUV) was adopted and furtherly quantified in the prefrontal cortex, the temporal cortex and the hippocampus, respectively. The SUV of apoE3-TR and apoE4-TR mice treated by FST stress decreased in both the 3-month-old and the 8-month-old groups. Of note, in both age groups (3 months and 8 months), under the same FST pressure, the SUV declines in the prefrontal cortex (Fig. 2B), temporal cortex (Fig. 2C) and hippocampus (Fig. 2D) of apoE4-TR mice were more significant than those of apoE3-TR mice. More surprisingly, when not receiving FST, the SUV of the 8-month-old apoE4-TR mice was obviously lower than that of the age-matched apoE3-TR mice. The above results indicate that apoE4-TR mice show age-dependent declines in cerebral glucose metabolism, which is further significantly exacerbated under acute stress. We infer that this decrease in glucose metabolism may be related to susceptibility to depression-like behaviors in the apoE4-TR mice.

### Exogenous ATP supplementation rescues the depression-like behaviors in the elderly apoE4-TR mice

**Fig. 3:**
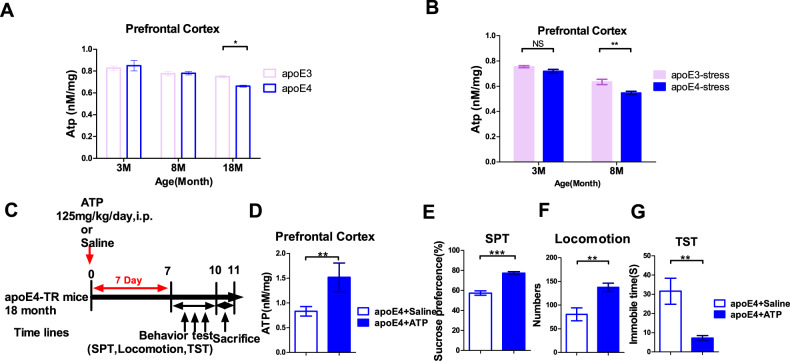
The rescued depression-like behaviors in elderly apoE4-TR mice by ATP supplementation. **A** ATP levels in the prefrontal cortex of apoE3-TR and apoE4-TR mice at 3, 8, and 18 months of age. *p < 0.05, by two-way ANOVA with Tukey’s Multiple Comparisons Test (*n* = 8–13). **B** ATP levels in the prefrontal cortex of apoE3-TR and apoE4-TR mice after FST at 3, 8 months of age. **p < 0.01, by two-way ANOVA with Tukey’s Multiple Comparisons Test (*n* = 9). **C** The time line of ATP administration and behavioral test. **D** ATP levels in the prefrontal cortex after ATP administration. For the prefrontal cortex. **p < 0.01, by two-tailed Student’s *t* tests (*n* = 6–7). Depression-like behavior measurements of 18-month-old apoE4-TR mice after ATP administration, including sucrose consumption percentage in SPT (**E**), locomotion in the open field (**F**), immobility time in the TST (**G**). **E**, **F** **p < 0.01, ***p < 0.001, by two-tailed Student’s *t* tests; **G** **p < 0.01, by Mann–Whitney test (*n* = 10). NS not significant (*p* > 0.05). Data are presented as the mean ± S.E.M.

The brain is a highly energy-consuming organ, demanding 20 times more energy than the rest of the body, and is highly sensitive to the damage to energy production. Previous studies have reported that ATP decline is involved in the biological mechanism of MDD [24, 31]. In the brain, the main source of ATP is the aerobic glycolysis. PET imaging showed that after stress or with aging, the glucose metabolism of apoE4-TR mice was significantly reduced when compared with the apoE3-TR mice. Therefore, the concentration of ATP in the prefrontal cortex of apoE-TR mice was investigated. As expected, the ATP in the PFC of the 18-month-old apoE4-TR mice was significantly lower than that of age-matched apoE3-TR mice (Fig. 3A). Moreover, the ATP concentration of the 8-month-old apoE4-TR mice significantly decreased when compared with the age-matched apoE3-TR mice after stress (Fig. 3B). Thus, we speculated that age-dependent or stress-induced reduction of ATP levels in the PFC may mediate depression-like behaviors in the apoE4-TR mice. To confirm the above hypothesis, we conducted a 7-day ATP (125 mg/kg/d, i.p.) supplementation on the elderly apoE4-TR mice, with the experimental procedure shown in Fig. 3C. We found that the level of ATP in the PFC of apoE4-TR mice significantly increased after the administration of ATP when compared with the normal saline treatment group (Fig. 3D). Subsequently, the behavior test revealed that ATP supplementation significantly increased the sucrose consumption (Fig. 3E), reduced the immobility time in the TST experiment (Fig. 3G) and enhanced locomotion in the open field (Fig. 3F) in the apoE4-TR mice. Taken together, the results suggest that regardless of aging or stress scenarios, the decline in ATP levels in the PFC contributes to the depression-like behaviors of the apoE4-TR mice, highlighting that the supplementation of ATP has a significant antidepressant-like effect in the apoE4-TR mice.

### *APOE* ε4 allele shows a culture-time-dependent damage to mitochondrial respiration and glycolysis of primary astrocytes

**Fig. 4:**
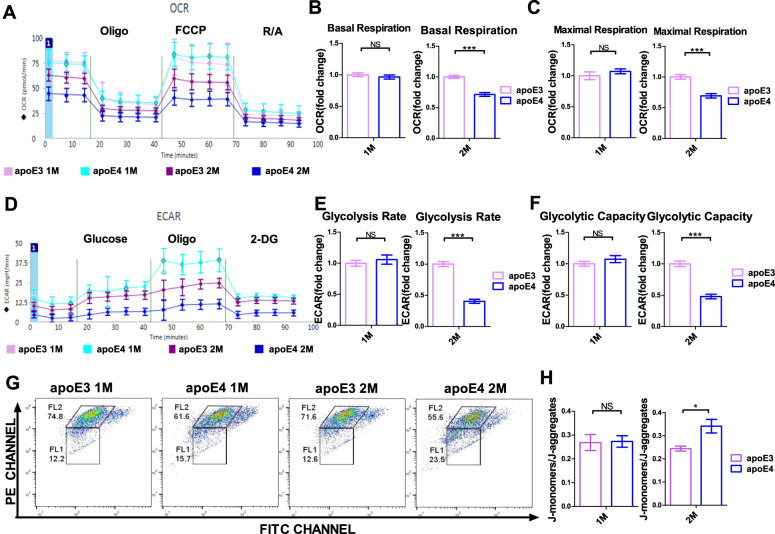
The disrupted glucose metabolism in primary astrocytes by *APOE* ε4 allele in a culture-time-dependent manner. **A** Bioenergy curves of changes in the rate of oxygen consumption (OCR) over time after the injection of various compounds that inhibited key enzymes in the mitochondrial electron transport chain. **B** The basal respiration of primary astrocytes from apoE3-TR and apoE4-TR mice after 1 and 2 months of culture. ***p < 0.001, by two-tailed Student’s *t* tests (*N* = 12–16). **C** The maximal respiration of primary astrocytes from apoE3-TR and apoE4-TR mice after 1 and 2 months of culture. ***p < 0.001, by two-tailed Student’s *t* tests (*N* = 12–16). **D** The change curve of extracellular acidification rate (ECAR) indirectly produced by glucose conversion to lactic acid during glycolysis. **E** The glycolysis rate of primary astrocytes from apoE3-TR and apoE4-TR mice after 1 and 2 months of culture. ***p < 0.001, by two-tailed Student’s *t* tests (*N* = 12–16). **F** The glycolytic capacity of primary astrocytes from apoE3-TR and apoE4-TR mice after 1 and 2 months of culture. ***p < 0.001, by two-tailed Student’s *t* tests (*N* = 12–16). **G** Representative flow cytometry analysis of JC-1 staining. Horizontal axis and vertical axis represent fluorescence intensities of JC-1 monomers and JC-1 aggregates, respectively. Increased intensity of monomers indicates decreased mitochondrial membrane potential. **H** The ratio of JC-1 monomers and aggregates is presented as the Mean ± S.E.M. of 3 independent experiments in each group. *p < 0.05, by two-tailed Student’s *t* tests (*N* = 3). NS, not significant (*p* > 0.05). Data are presented as the mean ± S.E.M.

Studies have shown that insufficient ATP release in astrocytes leads to depression-like behaviors, while stimulation of ATP release in astrocytes can bring about the antidepressant effect [24]. ApoE is mainly expressed in astrocytes in normal adult human brain. In order to further study the effect of different *APOE* genotypes on glucose metabolism of astrocytes, we evaluated the glucose metabolism of primary cultured astrocytes from the cortex of newborn apoE-TR mice with a Seahorse XF96 extracellular flux analyzer. Astrocytes from the cortex of apoE-TR mice were cultured for 1 and 2 months and re-inoculated on Seahorse XF96 plates at a density of 10,000 cells per well two days before the mitochondrial respiration test. The bioenergy curve was plotted according to the changes in the rate of oxygen consumption rate (OCR) assessed overtime after the injection of different inhibitory compounds of critical enzymes in the mitochondrial electron transport chain during the mitochondrial respiration test (Fig. 4A). In astrocytes cultured for one month, no significant changes in baseline and maximal mitochondrial respiration were observed between apoE3 and apoE4 (Fig. 4B, C). Interestingly, in apoE4-expressing astrocytes cultured for two months, the baseline and maximum mitochondrial respiration were significantly reduced when compared with that of apoE3-expressing astrocytes (Fig. 4B, C). These results suggest that with the extension of culture time, *APOE*4 damages the mitochondrial respiration in astrocytes.

Similarly, we evaluated the glycolysis of astrocytes with the Seahorse XF96 extracellular flux analyzer. Cortical astrocytes from apoE3-TR and apoE4-TR mice were re-inoculated on Seahorse XF96 plates at a density of 10,000 cells per well after one month and two months of primary culture. The glycolysis experiment was performed two days later. The curve in Fig. 4D was based on the change of extracellular acidification rate (ECAR) indirectly produced by the conversion of glucose to lactic acid during the glycolysis over time. Our data suggest that after one-month culture, the glycolytic rate and glycolytic capacity were not significantly changed between cortical astrocytes expressing apoE4 and those expressing apoE3 (Fig. 4E, F). However, after 2-month culture, the glycolytic rate and glycolytic capacity of astrocytes expressing apoE4 were significantly lower than those of astrocytes expressing apoE3 (Fig. 4E, F). Altogether, the results suggest that with the extension of culture time, *APOE*4 damages glycolysis in astrocytes and can impair the ATP production in the mitochondria.

Mitochondria are the main organelles that produce ATP. Changes in mitochondrial DNA and cell membrane permeability were found in both depression patients and animal models of depression [32, 33]. Available literature also shows that apoE4 (1-272) fragments can directly interact with mitochondria, causing mitochondrial dysfunction and neurotoxicity [13]. Therefore, JC-1 staining was used to detect mitochondrial membrane potential and investigate whether *APOE* genotypes had different effects on the mitochondrial function of primary astrocytes. Two months after culture, the ratio of JC-1 monomers and aggregates in the apoE4-expressing astrocytes were increased when compared with those of the apoE3-expressing counterparts (Fig. 4H). However, no similar findings were evident in the primary astrocytes after one-month culture (Fig. 4H). Increased intensity of monomers indicated decreased mitochondrial membrane potential. These results suggest that with the extension of culture time, *APOE*4 genotypes damage mitochondrial function in astrocytes.

## Discussion

In the present study, we confirmed that *APOE* ε4 allele exacerbated depression-like behaviors in mice during aging. Compared with the apoE3-TR mice, apoE4-TR mice showed significant declines in regional cerebral glucose metabolism and ATP level. Of note, ATP supplementation alleviated the depression-like behaviors in elderly apoE4-TR mice. Moreover, *APOE* ε4 damaged the mitochondrial respiration and glycolysis, and decreased mitochondrial membrane potential of primary astrocytes from apoE-TR mice. These findings provide insights into the role of *APOE4* as a risk factor for depression during aging and the underlying mechanism may involve the impaired glucose metabolism and ATP decline in astrocytes of apoE4-TR mice.

The relationship between *APOE* ε4 allele and depression has long been a research focus. However, due to limited sample size, wide disparities in study designs and procedures, there are approximately as many studies of *APOE* ε4 and depression that report positive results as those that find null results [2]. Our previous research has shown that compared with apoE3 at the age of 3 months, apoE4 did not aggravate the depression-like behaviors under chronic unpredictable mild stress (CUMS) [34]. In the current study, we demonstrated the potential role of the *APOE* ε4 allele in the development of depression during aging, which provides substantial evidence for the clinical relationship between *APOE* ε4 allele and late life depression (LLD) [35]. It is well known that *APOE4* is the strongest risk factor for late-onset Alzheimer’s disease [36]. Depression and AD are strongly associated. An early-life depression event elevates the subsequent AD risk [34]. Depressive symptoms may be direct manifestations of AD pathophysiology at the preclinical stage, prior to the onset of mild cognitive impairment [37–39]. Patients suffering from AD are more prone to comorbid depression [40, 41], and depressed people often have impaired cognition [42–44]. Taken together, *APOE4* is a major genetic risk factor for cognitive impairment and depression in the elderly population.

Research on *APOE4* has mainly focused on the neuronal damage caused by defective cholesterol transport [45] and exacerbated amyloid-β [46] and Tau pathology [47]. Recently, the impact of *APOE4* on astrocyte functions has attracted much attention. Studies have documented that astrocytic apoE4 increases tau-induced synaptic loss and microglia phagocytosis of synaptic elements [48], and that it is involved in the regulation of synaptic pruning, which suggests a key role for astrocytic apoE in synaptic integrity [49]. ApoE4 disrupts intracellular lipid homeostais [50] and diminishes neurotrophic function of human iPSC-derived astrocytes [51]. Ca hyperactivity associated with ApoE4 is found in astrocytes from targeted replacement mice [52]. ApoE4-expressing astrocytes alter mitochondrial dynamics and function [53] and affect glucose utilization, including increases in lactate synthesis, PPP flux, and de novo biosynthesis pathways [54]. In the present study, the long-term cultured astrocytes from apoE4-TR mice obviously decreased the mitochondrial membrane potential, mitochondrial respiration, and glycolysis. In conclusion, apoE4 directly affects many functions of astrocytes. In animal models of Alzheimer’s disease (AD), astrocytes undergo degeneration and atrophy at the early stages of pathological progression [55]. Similarly, astrocytic atrophy and decreased expression of the astrocyte biomarker GFAP have been documented in both animal depression models and autopsy specimens of depressed patients [25]. Also, studies have shown that insufficient ATP release in astrocytes leads to depression-like behaviors, while stimulation of ATP release in astrocytes produces an antidepressant effect [24]. Therefore, *APOE4*-induced astrocyte dysfunction may be the common pathophysiological basis of *APOE4*-mediated late-life depression and late-onset Alzheimer’s disease.

Accumulating evidence suggests that patients with major depressive disorder have decreased glucose metabolism in the prefrontal cortex (PFC), dorsal anterior cingulated cortex (dACC) [14]. Antidepressant effects of low-dose ketamine [56] and transcranial magnetic stimulation can improve the cerebral glucose metabolism in these regions [57–60]. In the present study, PET-CT images of [18F]-FDG uptake showed that when compared with *APOE* ε3 genotype, *APOE* ε4 genotype accelerated the decline of glucose metabolism in the frontal cortex and temporal cortex during aging and stress. Interestingly, in the case of acute stress, a statistical decrease in glucose metabolism was evident in the apoE4-TR mice at 3 months of age (Fig. 2B, C), but the significant difference in depression-like behaviors surfaced only when compared with apoE3-TR mice at 8 months of age (Fig. 1E). The results suggest that the decline in glucose metabolism predates the onset of depressive behaviors. Future research is needed to investigate if a drop in glucose metabolism can be an early warning of future depression.

Normally, glucose is the major energy source for the brain and is metabolized into ATP via glycolysis, the tricarboxylic acid (TCA) cycle and the electron transport chain (ETC). We found that ATP levels in the prefrontal cortex decreased in elderly or middle-aged stressed apoE4 mice when compared with the age-matched apoE3 mice, and that ATP supplementation significantly improved depression-like behaviors in the elderly apoE4 mice. Our data further validate that the dysregulation of extracellular ATP, a significant mediator of astrocyte-neuron communications [61, 62], is involved in depression-like disorders in rodents [24, 63, 64]. Astrocytes, the most abundant glial cells of the brain, play a vital role in the pathophysiology of depression [25] and secrete adenosine ATP, which modulates depression-like behaviors [65, 66]. Our research shows that the apoE4 disrupts the glucose metabolism of astrocytes in a culture-time-dependent manner, manifested by a decrease in the baseline and maximum mitochondrial respiration, and glycolysis rate, and glycolysis capacity. Of course, the long-term cultured astrocytes cannot replace those in aged brains. ApoE4 plays an age-dependent role in depression and cognitive decline, and future research is needed to explore the underlying mechanism by which ApoE4 influences astrocyte function. In general, a common design to use the same group of mice for multiple tasks is to allow 4–7 days rest between tests for minimizing carryover effects. In present study, elderly apoE4-TR mice were only treated with ATP for one week, and behavioral tests were performed as soon as possible to prevent drug failure. According to the principal of beginning with the test with minimized stress stimulation, each mouse was subjected to behavioral tests in the following order (Fig. 3): sucrose preference test, open field test, tail suspension test. Mice were rested in their home cage for 24 h between the later two behavior tests.

In summary, *APOE* ε4 allele increases the vulnerability to depression during aging and stress. ApoE4 mediates the declines in glucose metabolism and ATP level, which contributes to the depression-like phenotypes during aging. Furthermore, ATP treatment rescues the depression-like behaviors of elderly apoE4-TR mice, suggesting that ATP supplementation may serve as a possible therapeutic avenue for the elderly depression patients who carry *APOE* ε4.
