# Correction to: *APOE4* genotype exacerbates the depression-like behavior of mice during aging through ATP decline

**DOI:** 10.1038/s41398-021-01721-z

**Published:** 2021-11-28

**Authors:** Wenting Fang, Naian Xiao, Guirong Zeng, Daode Bi, Xiaoman Dai, Xue Mi, Qinyong Ye, Xiaochun Chen, Jing Zhang

**Affiliations:** 1 Department of Neurology and Geriatrics, Fujian Institute of Geriatrics, Fujian Medical University Union Hospital, 29 Xinquan Road, Fuzhou, Fujian 350001 China; 2 Fujian Key Laboratory of Molecular Neurology, Institute of Neuroscience, Fujian Medical University, 88 Jiaotong Road, Fuzhou, Fujian 350005 China; 3 Department of Neurology, The First Affiliated Hospital of Xiamen University, 55 Zhenhai Road, Xiamen, Fujian 361003 China

**Keywords:** Depression, Neuroscience

Correction to: *Translational Psychiatry* 10.1038/s41398-021-01631-0, published online 5 October 2021

The original version of this article unfortunately contained a mistake in Figure 2A. The authors apologize for the mistake. The original article has been corrected.

**Fig. 2:**
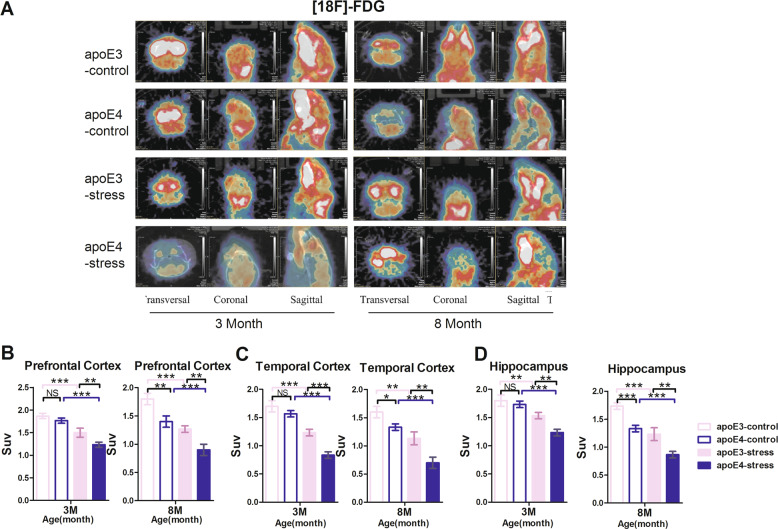
▉.

